# Perception of frontal facial images compared with their mirror images: chirality, enantiomorphic discrimination, and relevance to clinical practice

**DOI:** 10.1186/s40902-023-00396-4

**Published:** 2023-08-28

**Authors:** Zaid B. Al-Bitar, Ahmad M. Hamdan, Abedalrahman Shqaidef, Umberto Garagiola, Farhad B. Naini

**Affiliations:** 1 Department of Orthodontics and Pediatric Dentistry, School of Dentistry, The University of Jordan, Amman, Jordan; 2 College of Dental Medicine, University of Sharjah, Sharjah, UAE; 3 Department of Clinical Sciences, College of Dentistry, Ajman University, Ajman, UAE; 4 Maxillofacial and Odontostomatology Unit, School of Dentistry, University of Milan, Milan, Italy; 5 Kingston Hospital and Queen Mary’s Hospital Sidcup, King’s College Hospital NHS Foundation Trust, London, UK

**Keywords:** Face, Mirror images, Mirror-reversed, Selfie, Perception

## Abstract

**Background:**

What we think we see consists of models constructed in our brains, which may be constrained, limited and perhaps modified at a cerebral level. Patients may view their mirror image differently to how others and the clinical team view them. Understanding potential variations in perception between real and mirror images is important in clinical practice. The aims were to assess differences in self-perception between frontal facial and mirrored photographs, comparing the results with selfie photographs.

**Methods:**

Facial photographs were taken by one investigator under standardized conditions for preclinical and clinical students. Each student took a selfie photograph at rest and smiling using his/her smartphone. A mirror image was generated for each image. Each student was shown his/her original and mirror image, without being informed which was which. For each pair of images, students were asked to choose which photograph they perceived as more attractive. A set of photographs of a male volunteer was shown to all participants, to choose either the original or mirror image as the more attractive.

**Results:**

Most observers preferred the true image of the volunteer (*P* < 0.05), which may be evidence that most people prefer the true image of others, which is how they normally view them. Most observers preferred their own original photograph in frontal view at rest and smiling (*P* < 0.05), but preferred the mirror image of their selfie photograph at rest and smiling (*P* < 0.05).

**Conclusions:**

Significant differences in perceptions of attractiveness between true and mirror-reversed frontal and selfie images were found. Observers preferred their image the way they view themselves in a mirror. The selfie is how other people view an individual. If a selfie is flipped horizontally, that is how an individual sees themselves in a mirror. Most observers preferred the mirror image of their selfie, which is how they would view themselves in a mirror.

## Background

Visual perception refers to the apprehension of an object by the observer’s mind through their sense of sight. The process requires both the detection and interpretation of information from the object by the observer. The problem is that what we “see” is not always a perfect representation of the object being viewed. The concept of visual illusions demonstrates that, in reality, what we think we see in fact consists of models constructed in our brains, which may be constrained, limited, and perhaps modified at a cerebral level.

**Fig. 1:**
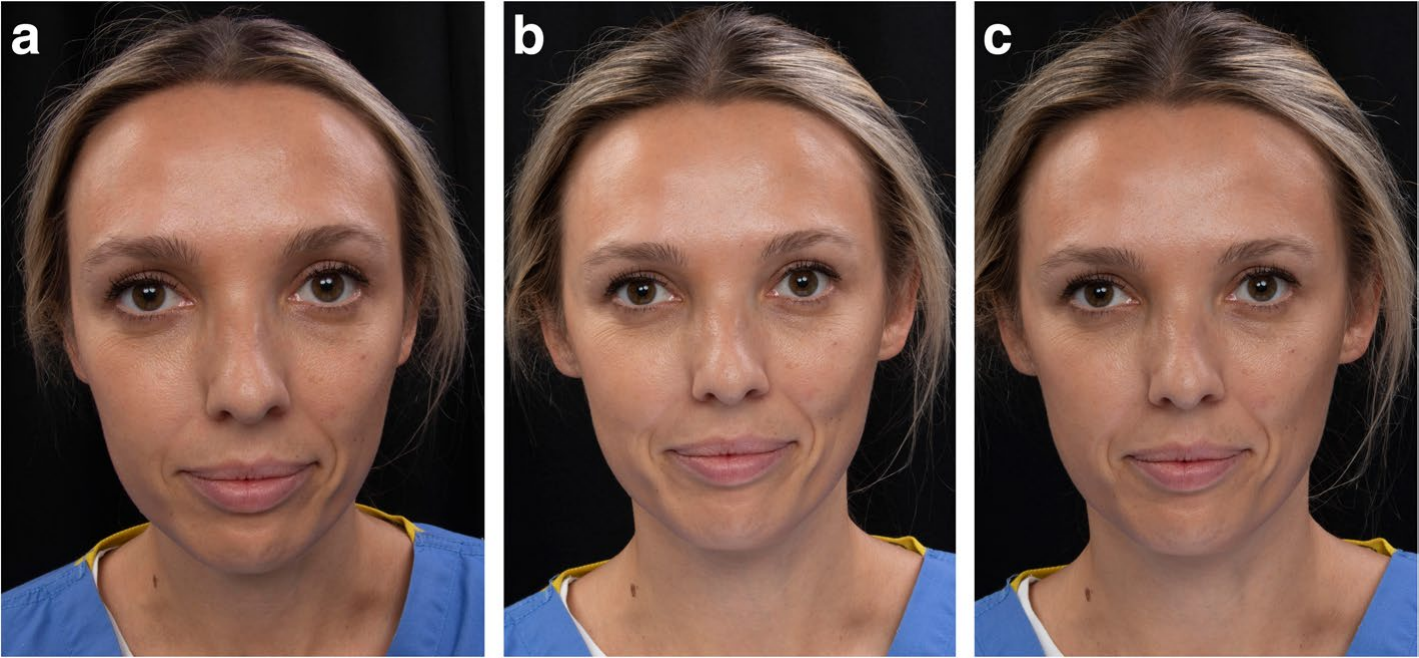
Photographs taken at different focal lengths: **a** 24 mm, **b** 90 mm, **c** 135 mm. Altering the focal length of the camera lens can change the resulting image. A camera with a 70–90 mm lens will provide the most “true to life” photograph of a patient

As craniofacial aesthetic evaluation in maxillofacial, plastic, and reconstructive surgery relies predominantly on the clinician’s ability to observe a patient accurately, understanding the process of visual perception and its limitations is of paramount importance. Moreover, clinicians rely heavily on the observation of clinical photographs of patients for diagnosis, planning and the analysis of treatment results. Simple variation in the focal length of a camera can alter and even distort facial photographs (Fig. 1). In addition, each patient views themselves in a mirror, whether pretreatment, preoperatively or postoperatively. This means that the patient views their face as a mirror image, thereby differently to how their family, friends, and the clinical team view them. As such, understanding potential variations in perception between real and mirror images is equally important. Most people spend their entire life only seeing their facial appearance as a mirror image reflection, sometimes referred to as a mirror-reversed image. They become used to seeing their face as a mirror image, and perhaps are more comfortable with their mirror image due to familiarity [1]. Often, their real image in a regular photograph taken by a standard camera, which is how others view them, may appear unusual to them, as they are so used to seeing their mirror image. Finally, the increased use of the “selfie”, a photograph that an individual takes of themselves, usually using their smartphone camera, is a substantial modern phenomenon with potentially negative repercussions. The selfie image on the smartphone camera roll is flipped horizontally, presumably because the manufacturers of the technology want the individual to see themselves as a mirror image, perhaps as that is what they would be used to seeing. Therefore, how the patient views themselves in a mirror or on a smartphone selfie is a mirror image, not how others will view them.

Symmetry may be defined as correspondence in size, shape and relative position of parts on either side of a dividing line or median plane. Asymmetry is defined as a lack or absence of symmetry [2]. Chirality, derived from the Greek word for “hand”, is a property of asymmetry. An object may be described as chiral if it is distinguishable from its mirror image in that it cannot be superimposed onto it, e.g. the left and right hands exhibit chirality. Chiral objects and their mirror images are referred to as enantiomorphic, derived from the Greek, literally meaning “opposite morphology”. The mirror image of a human face exhibits chirality and enantiomorphism. Specifically, the real facial appearance and its mirror image cannot be superimposed, i.e. they are not the same as one another.

It may be speculated that for many people, the image of themselves that they are least used to seeing, whether real or mirror, may intensify and perhaps exaggerate their least favorite features, or the features that they view as imperfections. For example, a 3-mm nasal tip asymmetry to the left in a real photograph will be viewed in a mirror image as a 3-mm asymmetry to the right, a significant visual difference of 6 mm.

The aims of this preliminary investigation were to assess whether there is a difference in self-perception of attractiveness between frontal facial and mirror image photographs, and to compare the results with selfie photographs.

## Methods

Ethical approval for the investigation was obtained from the Research and Ethics Committee at the University of Jordan. Consent forms explaining the nature and goals of the study were sent to all the participating dental students at the School of Dentistry.

**Fig. 2:**
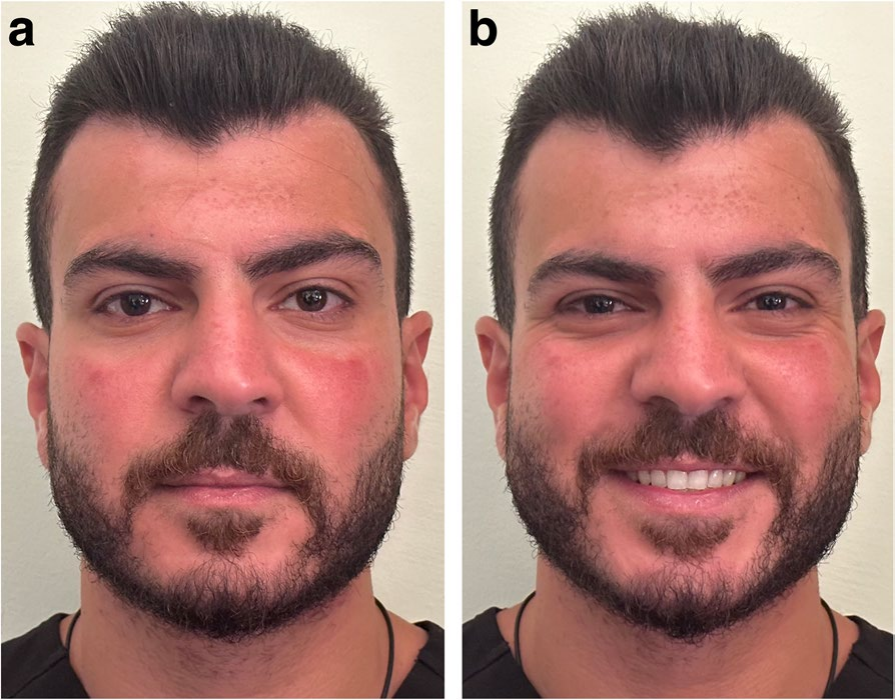
Facial photograph of a participant **a** at rest, and **b** smiling

**Fig. 3:**
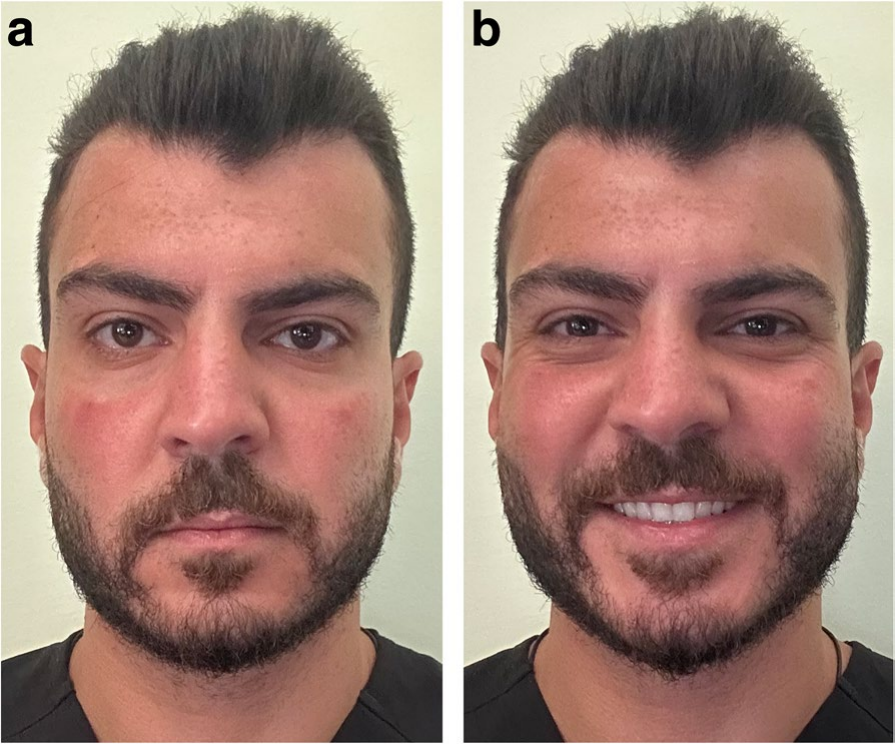
Selfie photograph of a participant **a** at rest, and **b** smiling

Facial photographs were taken by one investigator under standardized conditions for preclinical and clinical year students who agreed to take part in the study. The photographs taken were frontal view at rest and frontal view with posed smile (Fig. 2). Each student was also asked to take a selfie photograph of him/herself at rest and smiling using his/her smartphone (Fig. 3).

During the same session, photographs were downloaded to a laptop with a 17-inch screen, and a mirror image was generated for each image using Adobe Photoshop® software. Each participant was shown his/her own original and a mirror image, without being informed which was the original and which the mirror image. For each pair of images, participants were asked to choose which photograph they perceived as more attractive or whether they had the same preference.

In addition to this, a set of photographs taken of a male volunteer was shown to all the participants and they were asked to choose which image, original or mirror image (the participants did not know which was which), they found to be more attractive or whether they had the same preference.

### Statistical analysis

Statistical analysis to determine the effect of gender and stage of study (preclinical or clinical) was carried out using a one-way ANOVA. Chi-squared tests were used to assess differences in participants’ preferences for original and mirror images for all the photographs in the study. Finally, Wilcoxon signed ranks tests were used to compare preferences of participants for standard and selfie frontal views at rest and when smiling. Significant differences were set at the 5% level (*P* < 0.05).

## Results

The total number of participants in the study was 198 (56% males and 44% females). Students were almost equally divided among the preclinical and clinical years (48.5% and 51.5% respectively).

There were no significant gender differences between participants for all photographs (*P* > 0.05), except for the frontal at rest photograph of the volunteer; 75 females and 87 males preferred the original image, 12 females and 17 males preferred the mirror image, while none of the females and 7 males had the same preference for both photographs (Table 1, *P* < 0.05).

**Table 1:** Assessment of observers’ preference for photographs of the volunteer

	Original photograph	Mirror image	Same preference
Frontal view at rest^a^	162 (81.8%)	29 (14.6%)	7 (3.5%)
Frontal view while smiling^b^	141 (71.2%)	40 (20.2%)	17 (8.6%)
Selfie at rest	141 (71.2%)	39 (19.7%)	18 (9.1%)
Selfie while smiling	101 (51%)	63 (31.8%)	34 (17.2%)

^a^Gender differences were significant *P* < 0.05

^b^Level of study (preclinical/clinical) differences were significant *P* < 0.05

There were no significant differences between assessments of preclinical and clinical students for all photographs (*P* > 0.05), except for the selfie at rest photograph of the volunteer; 73 preclinical and 68 clinical students preferred the original photograph, 19 preclinical and 21 clinical students preferred the mirror image, while 4 preclinical and 13 clinical students had the same preference for both photographs (Table 1, *P* < 0.05). Chi-squared tests showed that there were significant differences between participants’ preferences of original and mirror images for all photographs included in the study (*P* < 0.05).

The majority of students preferred the original photograph over the mirror image for views taken of the volunteer, while only a minority of students had the same preference for both photographs (Table 1). A comparison of preferences of photographs for frontal view at rest and selfie at rest yielded significant differences (*P* < 0.05). Similarly, significant differences were found between preferences of photographs of frontal view while smiling and selfie while smiling (*P* < 0.05).

The majority of participants preferred their own original photograph in frontal view at rest (59.1%) and while smiling (58.6%). Conversely, most participants preferred the mirror image of their selfie photograph at rest (56.6%) and while smiling (50.5%, Table 2). There was only a minority of participants who had the same preference for both their own original and mirror image photographs (Table 2). A comparison of preferences of participants’ own photographs in frontal view at rest and selfie at rest yielded significant differences (*P* < 0.05). Similarly, significant differences were found between preferences of photographs of frontal view while smiling and selfie while smiling (*P* < 0.05).

**Table 2:** Assessment of observers’ preference for their own photograph

	Original photograph	Mirror image	Same preference
Frontal view at rest	117 (59.1%)	69 (34.8%)	12 (6.1%)
Frontal view while smiling	116 (58.6%)	70 (35.4%)	12 (6.1%)
Selfie at rest	61 (30.8%)	112 (56.6%)	25 (12.6%)
Selfie while smiling	71 (35.9%)	100 (50.5%)	27 (13.6%)

*P* < 0.05

## Discussion

The earliest self-reflected mirror images of humans are likely to have been reflections in pools of still water, which is alluded to in the Greek myth of Narcissus falling in love with his own reflection. Manufactured mirrors may date back to approximately 6000 BC, made from naturally occurring volcanic glass. Therefore, over thousands of years, humans have become accustomed to seeing their own face as a mirror image reflected to them, rather than their real appearance that is observed by others. The increased use of smartphone cameras and selfie images over the past decade may have increased the tendency of people viewing their mirror images, often of their facial appearance.

The results of the present investigation found no significant gender differences between observers for any photographs except the frontal at rest photograph of the volunteer. For this individual’s set of photographs, the majority of observers preferred the original true image, with only a small number showing preference for the mirror image, and an even smaller number having the same preference. This is evidence that most people appear to prefer the true image of others, which is how they normally view them. Stage of clinical training for the observers did not appear to be a significant determining variable. The majority of observers preferred their own original photograph in frontal view at rest and smiling. Conversely, most observers preferred the mirror image of their selfie photograph at rest and while smiling, with only a minority having the same preference for both their own original and mirror image photographs. Differences in perception were also found between the observers’ own photographs in frontal view at rest and selfie at rest, and between preferences of photographs of frontal view while smiling and selfie while smiling. The observers appear to prefer their image the way they view themselves in a mirror. The selfie is how other people view an individual. If a selfie is flipped horizontally, that is how an individual sees themselves in a mirror. Therefore, it is logical that most observers preferred the mirror image of their selfie, which is how they would see themselves in a mirror.

The results of this investigation may be relevant to multiple specialties in medicine, surgery and the social sciences. For example, Felig and Goldenberg [3] investigated the phenomenon of selfie-evaluation and behavior. In an extensive meta-analysis of the available literature, they found inconsistent evidence in the relationship between selfie behaviors and appearance-specific self-evaluations. However, they did find that selfie editing was related to negative self-evaluations both generally and specific to appearance. It may be that time spent editing an image of oneself may be indicative of an underlying psychosocial issue, although this is purely speculative and requires further investigation.

Mombaerts and Missotten [4] described a case series of patients with an ocular prosthesis and their mirror images. They found that of the 16 patients with an ocular prosthesis, all preferred their mirror image. The authors acknowledged that family and friends often prefer the real image of the patient with an ocular prosthesis to the mirror image. De Runz et al. [5] looked at female plastic surgery patients, with particular emphasis on facial aesthetic surgery, in relation to preferences for standard photographs compared with mirror images. They acknowledged that familiarity with an image, e.g. a mirror image of themselves, may lead to patients preferring that image rather than their real image, as viewed by others or in a standard photograph. Faces are asymmetrical, so faces in photographs are different from those observed in mirrors. The main objective of this study was to assess whether female plastic surgery patients preferred standard photographs or mirror-reversed photographs of themselves. They found that of 214 patients, predominantly middle-aged, 73% preferred their mirror-reversed photographs, a phenomenon that was more pronounced among patients undergoing facial aesthetic surgery.

Pouwels et al. [6] looked at potential differences in aesthetic appreciation of a left peripheral facial palsy compared to a right peripheral facial palsy, in relation to true or mirror images. Pictures of patients with a facial palsy were reversed as a mirror image and shown as a pair together with the true image to 42 patients and 24 medical professionals familiar with facial palsy. The observers were asked to choose the most attractive photograph. One of the aims was to assess the preferences for the mirror or true images. When comparing mirror and true image, 90% of patients with a left-sided facial palsy chose their mirror image as most attractive, compared with only 30% of patients with a right-sided facial palsy. Medical professionals found a right-sided facial palsy cosmetically less attractive than left-sided. Patients, especially with a left-sided facial palsy, tended to choose their mirror image, although this choice seems to be influenced by hemispheric specialization and, interestingly, with familiarity. Vision is a physiological process. The eyes do not “see”, but detect light, allowing the perceiver’s brain to construct a visual representation of the viewed object. What we see is, to all intents and purposes, a mind-generated image of the real object. These results demonstrate that our understanding of how the brain analyses images, whether true or mirrored, requires further investigation.

Another area of clinical practice where distortions in photographs may be potentially significant is in relation to body image disorders [7]. Body image disorders, such as body dysmorphic disorder (BDD), and eating disorders related to disturbances in body image perception, such as anorexia nervosa and bulimia nervosa, have in common dissatisfaction with self-appearance, and a link between perception of appearance and feelings of low self-worth. The distortions in body image affect the individual’s self-esteem. Patients with BDD or the aforementioned eating disorders tend to spend an inordinate amount of time observing themselves in a mirror or in photographs [8], which in the age of smartphones is more likely to be with selfie photographs. Body image disturbances may elevate to delusionality, which may be related at least in part to perceptual aberrations and problems with visual processing [9]. It has been proposed that the negative body image perception in these conditions may be related to deficits in visual perception, with evidence particularly related to facial appearance, where it has been found that patients with BDD and anorexia nervosa demonstrate deficits in holistic visual processing (difficulty in seeing the big picture) but exaggerated visual processing in relation to small details [10]. Both groups have been assessed using functional magnetic resonance imaging and electroencephalography in relation to visual processing, and both have shown deficits in relation to facial visual processing [11]. One of the methods currently employed in cognitive behavior therapy for patients with BDD involves what is termed perceptual retraining, with which the patient looks at his or her mirror reflection, describing their image in a non-judgemental way. The intention is to draw attention away from obsessive attention to minor details in appearance [12]. As this therapy is related to mirror image assessments, it is important that evaluations related to assessments of mirror images compared to real images (e.g. standard photographs) are better understood [13, 14]. The potential relationship between visual processing disorders, body image disturbances, and self-evaluation of real and mirror images requires evaluation.

A potential limitation of this investigation was that the methodology was limited to static images, whereas it should be borne in mind that individuals observing themselves in a mirror may be in a dynamic state, e.g. smiling or possibly speech. During the dynamic process of speech, it has been observed that over three-quarters of people demonstrate greater amplitude of movement on one side of the mouth compared with the other [15]. Additionally, it would be useful to compare responses from a lay population, as the participants in this investigation were dental students.

## Conclusions

There are significant differences in perceptions of attractiveness between true and mirror-reversed frontal images and selfie images. Clinicians in any specialty requiring facial photographs of patients should be aware of these issues, both for their own diagnostic and planning endeavors, and in relation to discussions with patients about self-assessment of their images. This is an area of research where further investigation is required linking visual processing and neurocognition with the reality of the use of photographs both in clinical practice and in the daily lives of patients.

## Data Availability

The datasets used and/or analyzed during the current study are available from the corresponding author on reasonable request.

## Abbreviation

BDD: Body dysmorphic disorder
